# Regulated Necrosis in HeLa Cells Induced by ZnPc Photodynamic Treatment: A New Nuclear Morphology

**DOI:** 10.3390/ijms151222772

**Published:** 2014-12-09

**Authors:** Jorge Soriano, Angeles Villanueva, Juan Carlos Stockert, Magdalena Cañete

**Affiliations:** Department of Biology, University Autonomous of Madrid, 28049 Madrid, Spain; E-Mails: jorge.soriano@uam.es (J.S.); angeles.villanueva@uam.es (A.V.); juancarlos.stockert@uam.es (J.C.S.)

**Keywords:** photodynamic therapy, photosensitizer, apoptosis, necrosis, regulated necrosis

## Abstract

Photodynamic therapy (PDT) is a cancer treatment modality based on the administration of a photosensitizer (PS), which accumulates preferentially in tumor cells. Subsequent irradiation of the neoplastic area triggers a cascade of photochemical reactions that leads to the formation of highly reactive oxygen species responsible for cell inactivation. Photodynamic treatments* in vitro* are performed with the PS, zinc-phthalocyanine (ZnPc). The PS is near the plasma membrane during uptake and internalization. Inactivation clearly occurs by a necrotic process, manifested by nuclear pyknosis, negative TUNEL and Annexin V assays and non-relocation of cytochrome c. In contrast, by increasing the incubation time, ZnPc is accumulated in the Golgi apparatus and produces cell inactivation with characteristics of apoptosis and necrosis: TUNEL positive, relocated cytochrome c and negative Annexin V assay. This type of death produces a still undescribed granulated nuclear morphology, which is different from that of necrosis or apoptosis. This morphology is inhibited by necrostatin-1, a specific inhibitor of regulated necrosis.

## 1. Introduction

Photodynamic therapy (PDT) is a cancer treatment modality based on the administration (systemic or topical) of a photosensitizer (PS) that accumulates preferentially in tumor cells [1,2,3]. Subsequent irradiation of the neoplastic area with red light triggers photochemical reactions, leading to the formation of highly reactive oxygen species (ROS, mainly singlet oxygen, ^1^O_2_), which are responsible for cell death [1,2,3,4]. PDT is also used in ophthalmology, dermatology, cardiology, rheumatology or for the treatment of infections [5,6,7].

Taking into account that the mechanisms involved in tumor destruction by PDT are complex and not completely established, cell cultures are an important tool to elucidate the precise process of cell photoinactivation.

Photodynamic treatments induce different mechanisms of cell death, such as apoptosis, necrosis, autophagy or mitotic catastrophe [8,9,10]. In PDT, necrosis or apoptosis is produced as a function of the PS, treatment doses, subcellular localization and cell type [11,12,13,14].

Traditionally, necrosis has been considered an unregulated process independent of apoptosis [15]. However, recent studies have demonstrated novel mechanisms with characteristics related to apoptosis and necrosis. These findings contradict the classic idea that apoptosis and necrosis are independent processes, and instead, they could have some signalizing pathways in common [16,17,18,19].

Considering the heterogeneity of the different routes, the nomenclature of these mechanisms of cell death is ambiguous (e.g., aponecrosis, necrapoptosis, caspase-independent cell death). The term “regulated necrosis” has been proposed in the last listing of the Nomenclature Committee on Cell Death to enclose these mechanisms, which occur in response to numerous damaging agents [19,20].

Furthermore, recent studies suggest that PDT with 5-aminolevulinic acid (5-ALA) is able to induce regulated necrosis in glioblastoma and osteosarcoma cells. In this case, the inductor stimulus is ^1^O_2_ originated in the mitochondria that provokes the formation of a receptor-interacting protein 3 (RIP-3) complex, although the mechanism by which ROS generate this response is still not known [21,22].

In several therapies and especially in oncology, a high degree of specificity to optimize the results and customize the protocols is required. Therefore, it is of great interest to know the action mechanism of a drug to allow improved protocols.

In this work, we have studied the influence of treatment time and subcellular localization of zinc-phthalocyanine (ZnPc) on the type of death mechanism triggered in HeLa cells. The results indicate that when the PS is observed in the Golgi apparatus (GA), the irradiation triggers a process of regulated necrosis, leading to a specific nuclear morphology different from that described for apoptosis or necrosis. On the contrary, when ZnPc is located in the plasma membrane [13], due to the internalization process, photodynamic treatments induce necrotic cell death.

## 2. Results

### 2.1. ZnPc Localization in HeLa Cells

**Figure 1:**
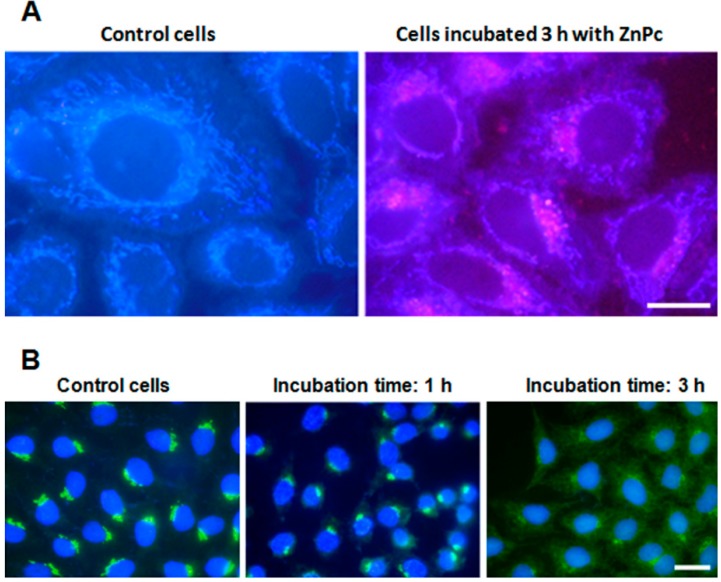
Zinc-phthalocyanine (ZnPc) localization in HeLa cells. (**A**) Control cells and cells incubated with 1 µM ZnPc for 3 h observed in fluorescence microscopy under UV excitation without any processing; (**B**) HeLa cells fixed immediately after photodynamic treatments, processed for indirect immunofluorescence for golgin-130 (GM130), counterstained with Hoechst-33258 (H-33258) and observed in fluorescence microscopy under UV and blue excitation light (overlay image). Scale bar: 20 µm.

After 1 h of incubation with ZnPc, it was not possible to locate PS inside cells, and only the blue autofluorescence of mitochondria was observed. By contrast, cells incubated with ZnPc for 3 h showed the red fluorescence of PS in a perinuclear position (Figure 1A).

Taking into account the localization of the PS, we evaluated the effect of photodynamic treatment on the GA by immunofluorescence of golgin-130 (GM130). Cells incubated with ZnPc for 1 h, irradiated and processed immediately later, presented a golgin-130-positive signal, located in the perinuclear position similar to the controls. Three hours after treatment, the GA structure still remains well organized. By contrast, cells incubated 3 h and processed immediately after irradiation showed a rapid disruption of the organelle, with a diffuse golgin-130 signal in the cytoplasm (Figure 1B).

### 2.2. Cell Survival

The survival rate of cells 24 h after treatments with ZnPc is observed in Table 1. In absence of irradiation, incubations with PS for 1 or 3 h do not affect cell viability, but after 10 min of irradiation, cells incubated 1 or 3 h with ZnPc showed a decrease in survival, near 100% in both cases.

**Table 1:** HeLa cell survival after different treatments, with and without irradiation. Data correspond to the mean values ± standard deviation (SD) from six different experiments.

Treatments	Dark Toxicity		10 min Irradiation Time (2.4 J/cm^2^)	
	Surviving Fraction (%)	SD	Surviving Fraction (%)	SD
Control	100	3.42	100	3.72
ZnPc 1 h	99.49	4.65	8.07	4.64
ZnPc 3 h	98.61	2.91	4.28	4.37

### 2.3. Nuclear Morphology

**Figure 2:**
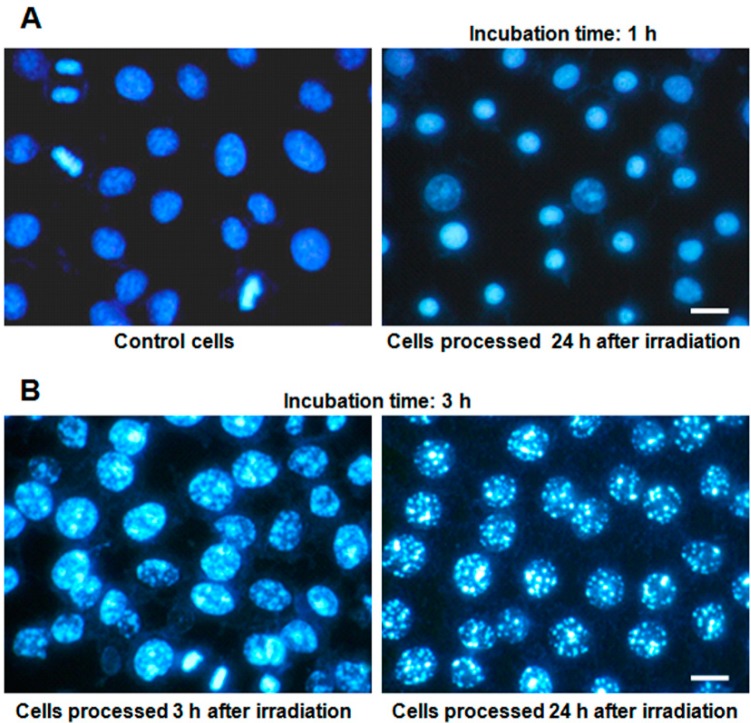
Nuclear morphology of H-33258-stained HeLa cells at different times after photodynamic treatments, observed in fluorescence microscopy under UV excitation light. (**A**) Control cells and cells treated with 1 µM ZnPc for 1 h, irradiated and processed 24 h later; (**B**) cells treated with 1 µM ZnPc for 3 h, irradiated and processed 3 and 24 h later. Scale bar: 20 µm.

The nuclear evolution of HeLa cells incubated with ZnPc, subsequently irradiated, and stained with Hoechst-33258 (H-33258) at different times after treatments is shown in Figure 2. Without irradiation, ZnPc-treated cells do not show morphological changes under any experimental conditions (Figure 2A). In contrast, most of the cells incubated 1 h with ZnPc and irradiated presented a small nucleus with highly condensed chromatin 1 h later, which clearly corresponds to a necrotic morphology (Figure 2A), which is maintained 24 h after irradiation.

Cells treated during 3 h with ZnPc, irradiated and observed 1 h later show chromatin condensation in the form of small spots, and 3 h after treatment, this condensation is evident (Figure 2B). Finally, 24 h after treatment, cells have nuclei larger than control cells, with highly condensed chromatin granules, variable in number and size, with the nuclear envelope apparently intact (Figure 2B). This morphology does not correspond to any classical apoptotic or necrotic processes. From now on, we will refer to these nuclei as “granulated nuclei”.

### 2.4. Characterization of Cell Death Type

**Figure 3:**
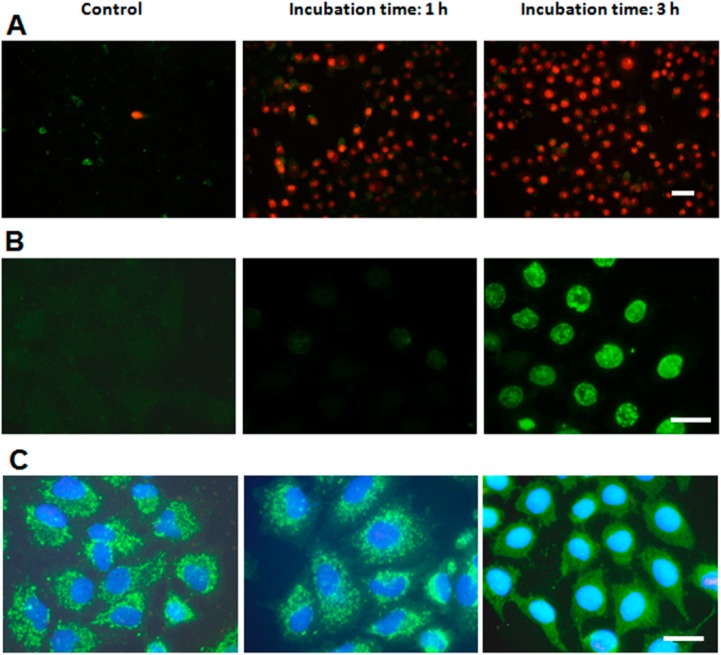
Assays to characterize the type of cell death performed at different times after photodynamic treatments. (**A**) HeLa cells incubated with 1 µM ZnPc for 1 and 3 h, processed for the Annexin V/propidium iodide (ANV/PI) assays immediately after irradiation and observed in fluorescence microscopy under blue and green excitation light (overlay); (**B**) HeLa cells subjected to the same treatments, processed for TUNEL assay 3 h later and observed in fluorescence microscopy under blue excitation; (**C**) HeLa cells incubated with 1 µM ZnPc (1 and 3 h), processed for indirect immunofluorescence for cyt-c immediately after irradiation, counterstained with H-33258 and observed in fluorescence microscopy under UV and blue excitation light (merged image). Scale bar: 20 µm.

Annexin V/propidium iodide (ANV/PI) assays are shown in Figure 3A. Control cells showed no positive signal for ANV or PI. Cells incubated with ZnPc for 1 h or 3 h and immediately processed after irradiation did not present positive staining for ANV, which indicates that no phosphatidylserine translocation occurred. On the other hand, most nuclei exhibited red PI fluorescence, due to loss of plasma membrane integrity.

A positive terminal deoxynucleotidyl transferase-mediated dUTP nick end-labeling (TUNEL) assay (Figure 3B) is a typical characteristic of apoptotic processes. Cells incubated 1 h with ZnPc, irradiated and processed 3 h later do not exhibit positive staining, showing images similar to control cells. On the contrary, cells incubated 3 h with ZnPc and processed 3 h after irradiation showed a strong nuclear labeling.

The translocation of cytochrome c (cyt-c) from mitochondria to the cytoplasm was also studied (Figure 3C). In control cells, cyt-c is located inside mitochondria, and cells treated 1 h with ZnPc and processed immediately after irradiation still retain cyt-c inside mitochondria. This situation continued 2 h after irradiation. By contrast, cells incubated with PS during 3 h and then irradiated exhibited cyt-c translocation immediately after irradiation, appearing as a green diffuse cytoplasm fluorescence that does not allow one to distinguish the mitochondrial morphology.

### 2.5. Treatments in Presence of Necrostatin-1

**Figure 4:**
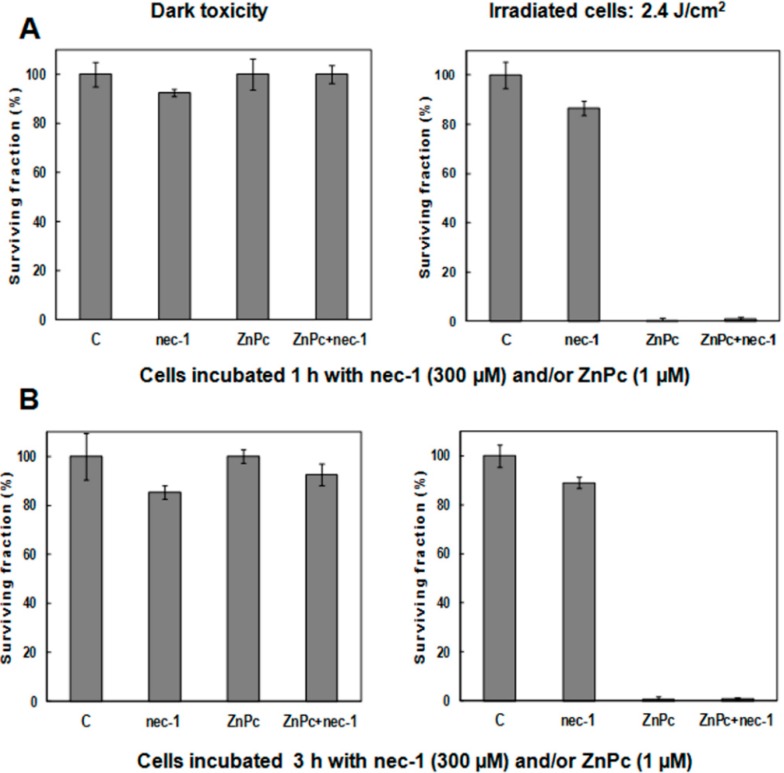
Survival of HeLa cells incubated with 1 µM ZnPc, 300 µM nec-1 or ZnPc − nec-1 and evaluated by the MTT assay 24 h after treatments. (**A**) Cells treated 1 h with ZnPc; (**B**) cells treated 3 h with ZnPc. Results represent the average of six independent experiments.

The effect of 300 µM nec-1, alone and/or in the presence of ZnPc, on HeLa cell survival is shown in Figure 4. Without irradiation, treatments with nec-1 and/or PS during 1 (Figure 4A) or 3 h (Figure 4B), do not alter cell viability significantly. After 10 min of irradiation or in the presence of ZnPc, HeLa cell survival is shown in Figure 4. After 10 min of irradiation time (Figure 4A,B), treatments with nec-1 do not affect cell survival.

In treatments with ZnPc + nec-1, the inhibitor does not alter the photodynamic effect of PS, producing nearly 100% cell inactivation with ZnPc incubations during 1 (Figure 4A) or 3 h (Figure 4B).

**Figure 5:**
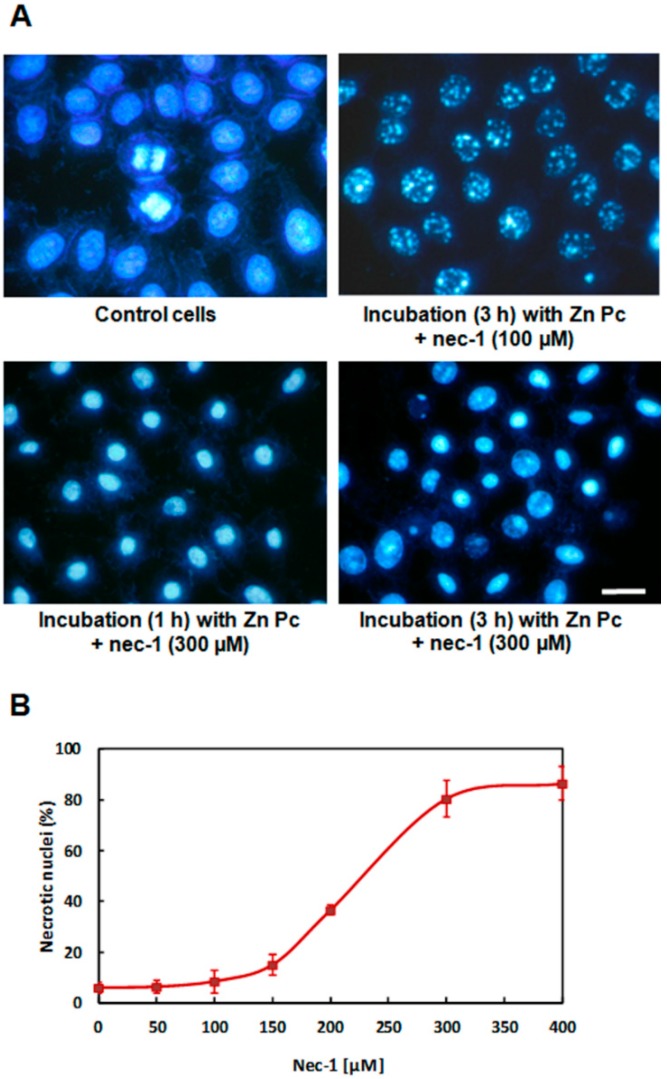
Effect of 300 µM nec-1 on the survival and nuclear morphology of HeLa cells incubated with 1 µM ZnPc (1 and 3 h) and evaluated 24 h after photodynamic treatment. (**A**) Cells stained with H-33258 and observed in fluorescence microscopy under UV excitation light; (**B**) the percentage of necrotic nuclei in HeLa cells incubated with 1 µM ZnPc for 3 h in the presence of different concentrations of nec-1, irradiated and then stained with H-33258 24 h after treatment. The morphological counting was carried out in fluorescence microscopy under UV exciting light. Each value is the average of six independent experiments. At least 2000 cells were counted in each experimental sample.

Morphological observations after photodynamic treatments in the presence of nec-1 were performed on cells stained with H-33258 24 h after treatments. Without irradiation, the nuclear morphology remained identical to that of controls (Figure 5). Irradiation of cells lacking the PS, but treated with nec-1 did not alter cellular morphology with any of the concentrations used. Cells incubated for 1 h with ZnPc in the presence of nec-1 (50–400 µM) and subsequently irradiated presented nuclei with necrotic morphology, identical to ZnPc phototreated cells. In contrast, photodynamic treatments using 3 h ZnPc + nec-1 incubation led to nuclear changes dependent on nec-1 concentration. Treatments with concentrations lower than 150 µM produced an identical morphology to that of cells treated only with the PS. However, cells treated with ZnPc + 150 µM nec-1 showed some necrotic nuclei. This effect was evident in cells incubated with ZnPc + 200 µM nec-1, where the amount of necrotic and granulated nuclei is equivalent. Finally, treatments with ZnPc + 300 or 400 µM nec-1 produced mostly necrotic nuclei (Figure 5A).

Figure 5B shows the increase in the number of necrotic nuclei produced by photodynamic treatments using different nec-1 concentrations. With concentrations lower than 150 µM, the number of cells with granulated nuclei is above 80%, but by increasing the nec-1 concentration, granulated nuclei decrease, and they are replaced by necrotic cells. With higher nec-1 concentrations (300–400 µM), the percentage of necrotic nuclei is above 80%. These results indicate that nec-1 has a concentration-dependent behavior, modifying significantly the type of cell death when administered at concentrations greater than 150 µM.

## 3. Discussion

Photodynamic treatments can induce different death mechanisms in cultured cells depending on experimental conditions. ZnPc phototreatments during 1 and 3 h of incubation time produce nearly 100% cell inactivation. However, from the morphological point of view, the cell death mechanism triggered in each case is different. PS incubation for 1 h produces a clear necrotic response, but incubation for 3 h causes cell death by a process characterized by the presence of granulated nuclei. Their formation can be observed 1 h after irradiation, remaining visible 24 h later, in a similar way as described for the tumor cell line, A-549 [23].

HeLa cells irradiated after 1 h PS incubation exhibited PI-stained nuclei without a positive signal for the ANV or TUNEL assays. These results are characteristic of typical necrosis and support the morphological studies.

By contrast, the TUNEL assay was positive in cells incubated with ZnPc for 3 h and processed 3 h after irradiation. However, with ANV/PI, the results were similar to those obtained with 1 h incubation, which is a characteristic of necrotic cell death. Changes in the type of cell death that depend on the incubation time appear to be related to the different PS location. A direct relationship exists in PDT between subcellular localization and the photodynamic effect, due to the short half-life of ^1^O_2_ and its limited diffusion capacity [24]. Consequently, the localization of PS and, therefore, the primary PDT target can be changed [25].

Using ZnPc incubation for 1 h, it is not possible to visualize the PS within the cell, because the amount of internalized ZnPc is not sufficient to be detected. With this incubation time and due to the internalization process, ZnPc was reported to be located at or mainly located in proximity of the plasma membrane [13], which rapidly loses its integrity and triggers a necrotic process. Although after 1 h of incubation with ZnPc, no red signal is detected on the cell surface, after 3 h, the PS is clearly accumulated in the GA. Therefore, it must be assumed that at shorter incubation times and below the sensitivity limit of microscopic detection, ZnPc is located at or near the plasma membrane before reaching the GA. Furthermore, the non-immediate alteration of the GA in phototreatments using 1 h of incubation with ZnPc confirms that the PS is not accumulated in this organelle. By contrast, after 3 h of incubation, the PS is located in a perinuclear position associated with the GA, as revealed by several authors [23,24,25,26]. Therefore, we think that under these experimental conditions, this organelle is the primary target of PDT, which is confirmed by its rapid damage. Cells incubated for 3 h with ZnPc, irradiated and processed for golgin-130 immunofluorescence showed immediate disruption of the GA, with a diffuse cytoplasmic staining. On the contrary, the GA signal remains localized in the perinuclear position at least 3 h after irradiation in samples incubated 1 h with the PS.

We also evaluated the role of mitochondria in the cell death induced by ZnPc. Mitochondrial membrane changes cause the release of cyt-c into the cytoplasm. Cyt-c forms the apoptosome, which activates caspase-9, triggering apoptotic processes [27]. However, some studies suggest that the release of other elements contained inside the mitochondria are involved in caspase-independent cell death [18,28,29,30].

Indirect immunofluorescence of cyt-c showed that photodynamic treatments with a 3 h ZnPc incubation produces the release of this protein in HeLa cells immediately after irradiation, as demonstrated by a diffuse cytoplasm signal. On the contrary, in samples treated with the PS for 1 h, cyt-c remains localized in the mitochondria at least 2 h after irradiation. In this case, the implication of this protein in early stages of cell death can be discarded.

We can conclude that Hela cells treated with ZnPc for 1 h show the characteristics of a typical necrotic process [31]. In contrast, the cell death mechanism that occurs with a 3 h ZnPc incubation has features of both apoptosis and necrosis. This process causes a new nuclear morphology, which is very different from apoptotic bodies and pyknosis of necrotic nuclei.

Among the different mechanisms of regulated necrosis, the most studied is that of necroptosis, which is triggered by the binding of some ligands to the TNFR family membrane receptors. In this case, cells show characteristics of apoptotic and necrotic processes. Necroptosis involves two kinases: RIP-1 and RIP-3. RIP-1 forms multimeric complexes with TNFR and other proteins. However, in the presence of certain stimuli, a deubiquitination process occurs in RIP-1, which binds to RIP-3 and forms the necrosome, triggering the necroptosis [32]. This binding can be repressed by nec-1, a compound that blocks the activity of RIP-1 kinase and that is considered a quite specific necroptosis inhibitor [33]. Once the necrosome has been formed, RIP-3 is able to interact with bioenergetic enzymes that generate an increase of ROS production, causing necroptosis [34,35]. Mitochondria would be also involved in this mechanism [36], but the integration of these processes is not yet known.

In the field of PDT, only a few works have dealt with the study of regulated necrosis in response to a photodynamic treatment. Results on glioblastoma cells treated with 5-ALA suggest that necroptosis can take place without the involvement of TNFR [21,22]. In this case, the inductor could be singlet oxygen produced by the photodynamic treatment, triggering necroptosis in response to intracellular stimuli. Taking into account that HeLa cells treated with ZnPc during 3 h presented characteristics associated with necroptosis, its possible involvement in photodynamic inactivation using nec-1 [33] as the inhibitor was assessed. Nec-1 caused a morphological change of nuclei in cells incubated 3 h with ZnPc and subsequently irradiated. In this case, a decrease of the number of granulated nuclei was observed, as well as an increase of necrotic nuclei. This effect was nec-1 concentration-dependent. It should be noted that nec-1 alone does not affect survival nor alter cell morphology under our experimental conditions. Cells incubated for 1 h with ZnPc and then irradiated did not show any alteration of the nuclear morphology in the presence of nec-1, and they showed near 100% inactivation by a necrotic process in all cases. Therefore, we think that necrosome activation is dependent on the precise subcellular location of ZnPc. These results indicate that the mechanism of cell death induced by a 3 h incubation with ZnPc corresponds to a regulated necrosis. The presence of nec-1 during photodynamic treatments prevents the formation of the necrosome and blocks the cascade of reactions that lead to a regulated necrosis displaying granular chromatin condensation. However, in these conditions, nec-1 does not avoid cell inactivation produced by photodynamic ROS generation, and cell death occurs by a necrotic process similar to what occurs with 1 h ZnPc treatments.

## 4. Experimental Section

### 4.1. Cell Culture

HeLa cells were grown in Dulbecco’s modified Eagle’s medium (DMEM) supplemented with 10% (*v*/*v*) fetal bovine serum (FBS), 50 units/mL penicillin and 50 μg/mL streptomycin. All products were provided by Invitrogen (Paisley, UK). Cell cultures were performed in a 5% CO_2_ atmosphere at 37 °C and maintained in a Steri-Cult 2000 incubator (Hucoa-Erloss, Madrid, Spain). The cells were seeded on 24-multiwell dishes (Falcon, St. Louis, MO, USA) with or without coverslips. Experiments were performed with cells at 60%–70% of confluence.

### 4.2. ZnPc Liposomes Preparation

Zinc(II)-phthalocyanine (ZnPc, Sigma–Aldrich, St. Louis, MO, USA) dissolved in pyridine was delivered in dipalmitoyl-phosphatidylcholine (DPPC, Sigma–Aldrich) liposomes [37]. ZnPc liposomes were sterilized through a Millipore^®^ filter (0.22 μm in diameter, Bedford, MA, USA) and diluted in DMEM without fetal bovine serum (FBS) to the appropriate concentration.

### 4.3. Subcellular Localization

Cells on coverslips were incubated for 1 or 3 h with 1 μM ZnPc. After incubation, cells were washed three times with PBS and directly observed under fluorescence microscopy (UV excitation) (see Section 4.12).

### 4.4. Photodynamic Treatments.

HeLa cells were incubated in serum-free medium with 1 μM ZnPc for 1 or 3 h. After treatments, cells were washed with PBS (×3) and maintained in whole medium during irradiation and post-treatment. Red light irradiation was performed using an LED source (λ = 640 ± 20 nm) with a mean intensity of 4 mW/cm^2^, measured with a M8 Spectrum Power Energy-meter (Merchantek Inc., San Diego, CA, USA), for 10 min (2.4 J/cm^2^).

### 4.5. Cell Viability

Cell viability was determined 24 h after treatments by the (3-[4,5-dimethylthiazol-2-yl]-2,5-diphenyltetrazolium bromide (MTT) assay (MTT, Sigma–Aldrich), according to current methods. Cell survival was expressed as the percentage of 540 nm absorption of treated cells in comparison with control cells. At least six independent experiments were carried out.

### 4.6. Golgin-130 Immunostaining

Cells were fixed in 3% paraformaldehyde (Panreac) for 15 min at 4 °C, washed three times in PBS and permeabilized with 0.1% (*v*/*v*) Triton X-100 in PBS for 5 min. Cells were incubated for 1 h with the primary mouse monoclonal GM130 antibody, (BD Transduction Lab, San Jose, CA, USA) at 37 °C, washed with PBS, permeabilized again with 0.1% (*v*/*v*) Triton X-100/PBS for 5 min, incubated with the secondary antibody (FITC-labeled goat anti-mouse IgG, Sigma–Aldrich) for 1 h at 37 °C and washed with PBS. Samples were counterstained with 5 μg/mL Hoechst 33258 (H-33258, Sigma–Aldrich), mounted in ProLong Gold (Molecular Probes, Carlsbad, CA, USA) and observed under fluorescence microscopy (UV and blue excitation).

### 4.7. Hoechst-33258 Staining

At different times after treatments, cells on coverslips were fixed in cold methanol for 5 min, air dried and stained with 5 μg/mL H-33258 for 2 min. Preparations were mounted in DePeX (Serva, Heidelberg, Germany) and observed by fluorescence microscopy under UV excitation. Well-known morphological criteria were used to assess necrotic and apoptotic cells [26,31].

### 4.8. Annexin V/Propidium Iodide Assay

Phosphatidylserine translocation was evaluated using ANV-fluorescein isothiocyanate (FITC, BD Biosciences, San Jose, CA, USA). Cells on coverslips were washed twice with PBS and once with ANV binding buffer. ANV-FITC (diluted 1:20 in ANV binding buffer) and PI (1 μg/mL) were then added to cells. After 15 min of incubation at room temperature in the dark, cells were washed twice with ANV binding buffer and observed under fluorescence microscopy (blue and green excitations).

### 4.9. Terminal Deoxynucleotidyl Transferase-Mediated dUTP Nick End-Labeling (TUNEL) Assay

To detect DNA fragmentation, the TUNEL assay was performed 3 h after the treatments. Cells were fixed in 3% paraformaldehyde for 15 min at 4 °C, washed with PBS (×3), permeabilized with 0.1% Triton X-100 in PBS for 2 min and incubated with TUNEL reaction mixture (Roche, Indianapolis, IN, USA) for 1 h at 37 °C. Cells were washed with PBS, mounted in ProLong Gold and observed under fluorescence microscopy (blue excitation).

### 4.10. Cytochrome c (Cyt-c) Immunostaining

Cells were fixed in 3% paraformaldehyde for 15 min at 4 °C, washed three times in PBS, permeabilized with 0.5% Triton X-100 in PBS for 5 min and incubated for 30 min with blocking solution (5% BSA, 5% FBS and 0.02% Triton X-100 in PBS) at room temperature. Cells were incubated with mouse monoclonal anti-cyt-c antibody (Invitrogen) for 1 h at 37 °C, washed in PBS and permeabilized again with 0.5% Triton X-100 in PBS for 5 min. Then, cells were incubated with the secondary antibody (FITC-labeled goat anti-mouse IgG, Sigma–Aldrich) at 37 °C for 1 h, washed with PBS, counterstained with H-33258 and mounted in ProLong Gold. Samples were observed under fluorescence microscopy (UV and blue excitation).

### 4.11. Necrostatin-1 (Nec-1) Treatments

Cells were incubated for 1 or 3 h in serum-deprived medium, with different concentrations (100–400 µM) of the known inhibitor of necroptosis [33], nec-1 (Sigma–Aldrich), in the presence or absence of 1 μM ZnPc. The samples were then washed with PBS (×3) and maintained in fresh complete medium.

### 4.12. Microscopy

Observations were performed with a BX61 epifluorescence microscope (Olympus, Hamburg, Germany), equipped with ultraviolet (UV, 365 nm), blue (450–490 nm) and green (510–550 nm) exciting filters. Photographs were taken with an Olympus DP50 digital camera and processed using Adobe Photoshop 8.0 (Adobe Systems, San Jose, CA, USA).

## 5. Conclusions

The present study shows that subtle differences in photodynamic treatments, particularly incubation times, can result in different morphological and signalizing cell death processes. Short-term PS incubation induces necrosis, whereas somewhat prolonged treatment results in a necroptotic response as a function of PS localization.

## References

[1] Juarranz A., Jaén P., Sanz-Rodríguez F., Cuevas J., González S. (2008). Photodynamic therapy of cancer. Basic principles and applications. Clin. Transl. Oncol..

[2] Agostinis P., Berg K., Cengel K.A., Foster T.H., Girotti A.W., Gollnick S.O., Hahn S.M., Hamblin M.R., Juzeniene A., Kessel D. (2011). Photodynamic therapy of cancer: An update. CA Cancer J. Clin..

[3] Lee Y., Baron E.D. (2011). Photodynamic therapy: Current evidence and applications in dermatology. Semin. Cutan. Med. Surg..

[4] Dolmans D.E., Fukumura D., Jain R.K. (2003). Photodynamic therapy for cancer. Nat. Rev. Cancer.

[5] Elizalde J., Vasquez L., Iyo F., Abengoechea S. (2012). Photodynamic therapy in the management of circumscribed choroidal hemangioma. Can. J. Ophthalmol..

[6] Waksman R., McEwan P.E., Moore T.I., Pakala R., Kolodgie F.D., Hellinga D.G., Seabron R.C., Rychnovsky S.J., Vasek J., Scott R.W. (2008). PhotoPoint photodynamic therapy promotes stabilization of atherosclerotic plaques and inhibits plaque progression. J. Am. Coll. Cardiol..

[7] St Denis T.G., Dai T., Izikson L., Astrakas C., Anderson R.R., Hamblin M.R., Tegos G.P. (2011). All you need is light: Antimicrobial photoinactivation as an evolving and emerging discovery strategy against infectious disease. Virulence.

[8] Rodriguez M.E., Zhang P., Azizuddin K., Delos Santos G.B., Chiu S.M., Xue L.Y., Berlin J.C., Peng X., Wu H., Lam M. (2009). Structural factors and mechanisms underlying the improved photodynamic cell killing with silicon phthalocyanine photosensitizers directed to lysosomes* versus* mitochondria. Photochem. Photobiol..

[9] Reiners J.J., Agostinis P., Berg K., Oleinick N.L., Kessel D. (2010). Assessing autophagy in the context of photodynamic therapy. Autophagy.

[10] Krammer B., Verwanger T. (2012). Molecular response to hypericin-induced photodamage. Curr. Med. Chem..

[11] Piette J., Volanti C., Vantieghem A., Matroule J.Y., Habraken Y., Agostinis P. (2003). Cell death and growth arrest in response to photodynamic therapy with membrane-bound photosensitizers. Biochem. Pharmacol..

[12] Castano A.P., Mroz P., Hamblin M.R. (2006). Photodynamic therapy and anti-tumour immunity. Nat. Rev. Cancer.

[13] Fabris C., Valduga G., Miotto G., Borsetto L., Jori G., Garbisa S., Reddi E. (2001). Photosensitization with zinc (II) phthalocyanine as a switch in the decision between apoptosis and necrosis. Cancer Res..

[14] Yoo J.O., Lim Y.C., Kim Y.M., Ha K.S. (2011). Differential cytotoxic responses to low- and high-dose photodynamic therapy in human gastric and bladder cancer cells. J. Cell. Biochem..

[15] Hail N., Carter B.Z., Konopleva M., Andreeff M. (2006). Apoptosis effector mechanisms: A requiem performed in different keys. Apoptosis.

[16] Formigli L., Papucci L., Tani A., Schiavone N., Tempestini A., Orlandini G.E., Capaccioli S., Orlandini S.Z. (2000). Aponecrosis: Morphological and biochemical exploration of a syncretic process of cell death sharing apoptosis and necrosis. J. Cell. Physiol..

[17] Asare N., Låg M., Lagadic-Gossmann D., Rissel M., Schwarze P., Holme J.A. (2009). 3-Nitrofluoranthene (3-NF) but not 3-aminofluoranthene (3-AF) elicits apoptosis as well as programmed necrosis in Hepa1c1c7 cells. Toxicology.

[18] Baritaud M., Boujrad H., Lorenzo H.K., Krantic S., Susin S.A. (2010). Histone H2AX: The missing link in AIF-mediated caspase independent programmed necrosis. Cell Cycle.

[19] Galluzzi L., Vitale I., Abrams J.M., Alnemri E.S., Baehrecke E.H., Blagosklonny M.V., Dawson T.M., Dawson V.L., El-Deiry W.S., Fulda S. (2012). Molecular definitions of cell death subroutines: Recommendations of the Nomenclature Committee on Cell Death. Cell Death. Differ..

[20] Vanlangenakker N., Berghe T.V., Vandenabeele P. (2012). Many stimuli pull the necrotic trigger, an overview. Cell Death. Differ..

[21] Coupienne I., Fettweis G., Piette J. (2011). RIP3 expression induces a death profile change in U2OS osteosarcoma cells after 5-ALA-PDT. Lasers Surg. Med..

[22] Coupienne I., Fettweis G., Rubio N., Agostinis P., Piette J. (2011). 5-ALA-PDT induces RIP3-dependent necrosis in glioblastoma. Photochem. Photobiol. Sci..

[23] Cristóbal J., Stockert J.C., Villanueva A., Rello-Varona S., Juarranz A., Cañete M. (2006). Caspase-2: A possible trigger of apoptosis induced in A-549 tumor cells by ZnPc photodynamic treatment. Int. J. Oncol..

[24] Moan J., Berg K. (1991). The photodegradation of porphyrins in cells can be used to estimate the lifetime of singlet oxygen. Photochem. Photobiol..

[25] Cañete M., Stockert J.C., Villanueva A. (2009). Preclinical photodynamic therapy research in Spain. 3. Localization of photosensitizers and mechanisms of cell death* in vitro*. J. Porphyr. Phthalocyanines.

[26] Rello-Varona S., Stockert J.C., Cañete M., Acedo P., Villanueva A. (2008). Mitotic catastrophe induced in HeLa cells by photodynamic treatment with Zn(II)-phthalocyanine. Int. J. Oncol..

[27] Mace P.D., Riedl S.J. (2010). Molecular cell death platforms and assemblies. Curr. Opin. Cell Biol..

[28] Johnson D.E. (2000). Noncaspase proteases in apoptosis. Leukemia.

[29] Kroemer G., Martin S.J. (2005). Caspase-independent cell death. Nat. Med..

[30] Moubarak R.S., Yuste V.J., Artus C., Bouharrour A., Greer P.A., Menissier-de Murcia J., Susin S.A. (2007). Sequential activation of poly(ADP-ribose) polymerase 1, calpains, and Bax is essential in apoptosis-inducing factor-mediated programmed necrosis. Mol. Cell. Biol..

[31] Rello S., Stockert J.C., Moreno V., Gámez A., Pacheco M., Juarranz A., Cañete M., Villanueva A. (2005). Morphological criteria to distinguish cell death induced by apoptotic and necrotic treatments. Apoptosis.

[32] Thapa R.J., Nogusa S., Chen P., Maki J.L., Lerro A., Andrake M., Rall G.F., Degterev A., Balachandran S. (2013). Interferon-induced RIP1/RIP3-mediated necrosis requires PKR and is licensed by FADD and caspases. PNAS.

[33] Xie T., Peng W., Liu Y., Yan C., Maki J., Degterev A., Yuan J., Shi Y. (2013). Structural basis of RIP1 inhibition by necrostatins. Structure.

[34] Lin Y., Choksi S., Shen H.M. (2004). Tumor necrosis factor-induced nonapoptotic cell death requires receptor-interacting protein-mediated cellular reactive oxygen species accumulation. J. Biol. Chem..

[35] Zhang D.W., Shao J., Lin J., Zhang N., Lu B.J., Lin S.C., . Dong M.Q., Han J. (2009). RIP3, an energy metabolism regulator that switches TNF-induced cell death from apoptosis to necrosis. Science.

[36] Karch J., Kwong J.Q., Burr A.R., Sargent M.A., Elrod J.W., Peixoto P.M., Martinez-Caballero S., Osinska H., Cheng E.H., Robbins J. (2013). Bax and Bak function as the outer membrane component of the mitochondrial permeability pore in regulating necrotic cell death in mice. Elife.

[37] Ginevra F., Biffanti S., Pagnan A., Biolo R., Reddi E., Jori G. (1990). Delivery of the tumour photosensitizer zinc(II)-phthalocyanine to serum proteins by different liposomes: Studies* in vitro* and* in vivo*. Cancer Lett..

